# Relationship of Neutrophil Percentage‐to‐Albumin Ratio With Stroke: Evidence From NHANES 1999–2020

**DOI:** 10.1002/brb3.70192

**Published:** 2024-12-22

**Authors:** Shuying Xu, Yiyan Sun, Siyao Zhang, Yongjun Peng, Dechun Dai, Leiyong Zhao

**Affiliations:** ^1^ Department of Acupuncture‐Moxibustion, Tuina and Rehabilitation Kunshan Hospital of Traditional Chinese Medicine SuZhou China; ^2^ College of Rehabilitation Medicine Shanghai University of Traditional Chinese Medicine Shanghai China; ^3^ Department of Treatment of Disease Kunshan Hospital of Traditional Chinese Medicine SuZhou China; ^4^ Department of Acupuncture and Rehabilitation Affiliated Hospital of Nanjing University of Chinese Medicine Nanjing China

**Keywords:** association, large‐scale, NHANES, NPAR, stroke

## Abstract

**Objective:**

The objective of this research is to examine the association between neutrophil percentage‐to‐albumin ratio (NPAR) and stroke, providing a reference for the prevention and prognosis of clinical stroke.

**Method:**

The study included 56,566 participants from the National Health and Nutrition Examination Survey (NHANES) to explore the association between NPAR and stroke using logistic regression and restricted cubic splines. Upon identifying the nonlinear relationship, inflection points were calculated using recursive algorithms and two‐stage linear regression models. Stratified analyses and interaction tests examined variations across population groups.

**Results:**

After adjusting for confounders, NPAR was positively associated with stroke (OR: 1.09, 95% CI: 1.05–1.12). Restricted cubic spline analysis indicated a nonlinear trend. Beyond the inflection point, the increasing trend of stroke incidence with higher NPAR levels began to slow down. This relationship remained nonlinear in males but was linear in females.

**Conclusion:**

This study revealed a nonlinear positive association between NPAR and stroke, with higher NPAR increasing the risk of stroke.

## Graphical abstract

This study identifies a nonlinear, positive association between the neutrophil percentage‐to‐albumin ratio (NPAR) and stroke risk. Higher NPAR values increase stroke incidence, with a sex‐specific correlation. NPAR may serve as a cost‐effective, accessible marker for early stroke risk detection, informing individualized prevention strategies in clinical and public health settings.

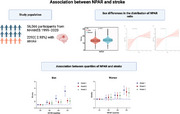

## 1 Introduction

Stroke is a major public health concern and one of the leading causes of mortality and disability worldwide, responsible for approximately 11% of all deaths globally (Feigin et al. 2022; Tsao et al. 2023). Although there have been advancements in medical interventions and preventive measures, the rising incidence and impact of stroke highlights the urgent need for a deeper understanding of its risk factors and underlying mechanisms (Tsao et al. 2023). In recent years, inflammation has gained recognition as a crucial element in the development of stroke. Numerous inflammatory markers have been examined for their prognostic significance in stroke, such as neutrophil counts and albumin levels (Macrez et al. 2011; Brooks et al. 2014).

The NPAR has become a promising biomarker by utilizing neutrophil counts and albumin levels, providing an affordable and easily accessible measure of systemic inflammation. It has shown promise in predicting outcomes in cardiovascular diseases, as well as in patients with acute kidney injury and cancer (Lv et al. 2023; Wu et al. 2023; Lessomo et al. 2023). Neutrophil‐driven inflammation plays a pivotal role in exacerbating post‐stroke injury by releasing proteolytic enzymes, reactive oxygen species (ROS), and pro‐inflammatory cytokines, leading to blood–brain barrier disruption, cerebral edema, and subsequent tissue damage (Silvestre‐Roig et al. 2020). In addition, neutrophils facilitate thrombus formation via neutrophil extracellular traps (NETs), further amplifying ischemic injury (Stowe et al. 2009). Albumin functions as a key antioxidant and vascular protector, with reduced albumin levels being associated with heightened oxidative stress and immune dysregulation, which may exacerbate neurological damage following stroke. Consequently, lower serum albumin levels heighten susceptibility to infectious complications (Thuemmler et al. 2024). Several studies have recognized albumin levels as an independent predictor of stroke recurrence. The dysregulation of the immunomodulatory roles of neutrophils and albumin can promote an excessive inflammatory response and thrombogenesis, thereby increasing stroke risk (Donkor 2018). Thus, NPAR may serve as a composite marker reflecting the interplay of systemic inflammation, thrombotic activity, and impaired antioxidant defenses, all of which contribute to stroke onset and progression. However, the specific association between NPAR and stroke incidence has not been thoroughly explored. This study aims to explore the association between NPAR and stroke using a large‐scale study from the National Health and Nutrition Examination Survey (NHANES) data spanning from 1999 to 2020.

## 2 Methods

### 2.1 Study Population

**FIGURE 1:**
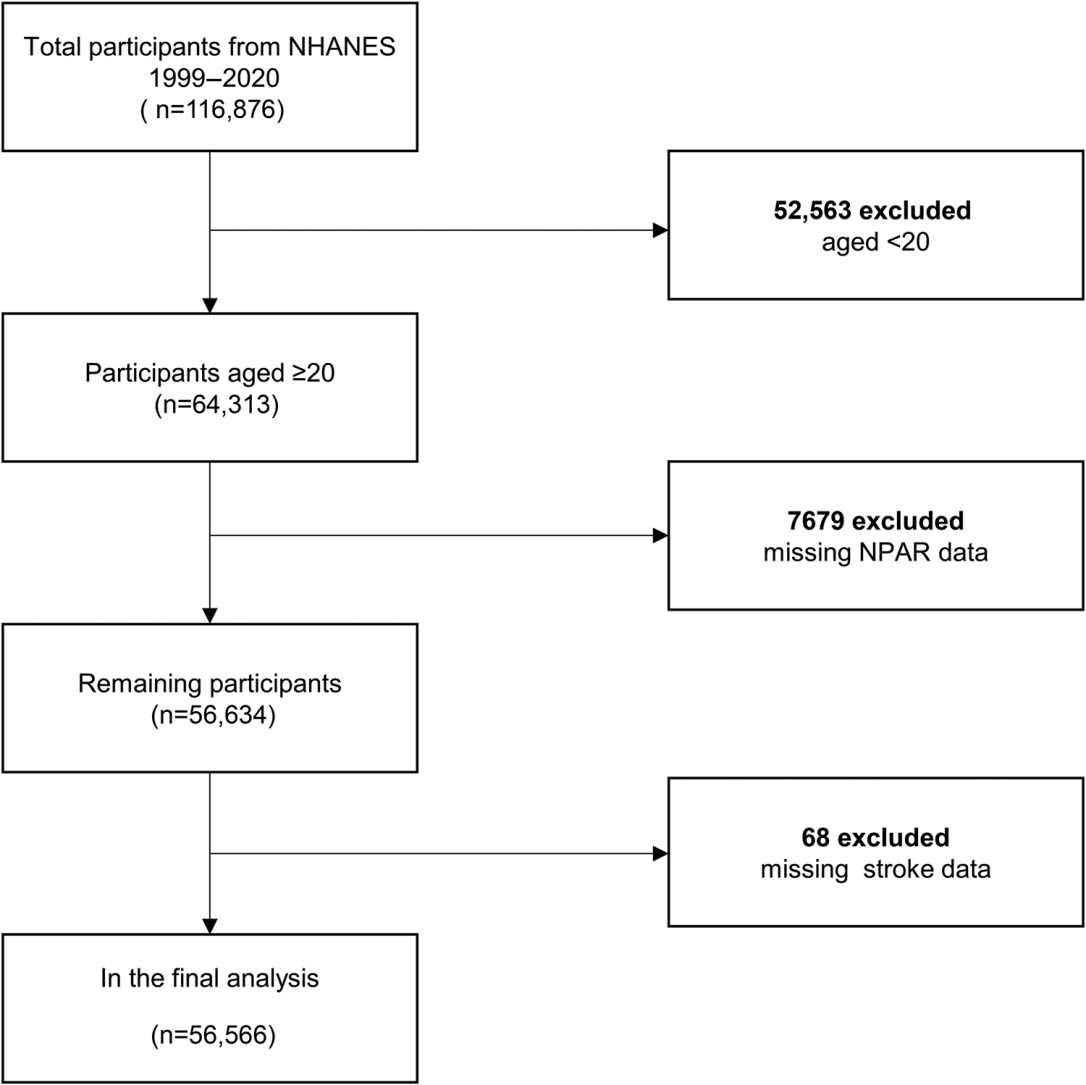
Flowchart depicting the selection process of participants from NHANES.

The NHANES database, conducted annually in the United States, includes both health interviews and physical examinations of its participants. It collects data on a two‐year cycle, examining 5,000 people across the United States each year at mobile testing centers. Weights are assigned to these participants to ensure that they are representative of the health and nutritional status of the general population in the United States. The Ethics Review Board of the National Center for Health Statistics has approved all data collected from NHANES participants (available on the web at: https://www.cdc.gov/nchs/nhanes/). All participants signed informed consent. **Figure** **1** illustrates the selection process for our study. From a total of 116,876 participants in the NHANES dataset from 1999 to 2020, individuals aged under 20 years (*n* = 52,563) were excluded. In addition, participants with missing NPAR data (*n* = 7,679) and missing stroke data (*n* = 68) were also excluded. Ultimately, data from 56,566 participants with complete information were utilized.

### 2.2 Assessment of NPAR

In the NHANES database, the evaluation of NPAR was conducted by extracting pertinent hematologic parameters from the complete blood count (CBC) profile. This included measuring neutrophil counts, lymphocyte counts, monocyte counts, neutrophil counts, white blood cell (WBC), and albumin levels. At the time of recruitment, a single blood sample was obtained from each participant. Comprehensive information on laboratory methodologies, quality assurance procedures, and data processing protocols is available on the NHANES website (https://wwwn.cdc.gov/Nchs/Nhanes/2013‐2014/CBC_H.htm). The Beckman Coulter method, utilizing an automatic dilution and mixing system, was employed for cell counting and sizing. To calculate NPAR, the neutrophil percentage was determined from the WBC count. This neutrophil percentage, combined with albumin levels from the same blood sample, was then used to compute NPAR using the following formula:

$$\mathrm{NPAR}\;=\frac{\mathrm{Neutrophil}\;\mathrm{percentage}\left(\%\right)}{\mathrm{Albumin}\left(g/\mathrm{dL}\right)}\;\times \;100$$

### 2.3 Assessment of Stroke

The diagnosis of stroke was determined through standardized medical condition surveys conducted via personal interviews. Specific questions included whether participants had been informed by health professionals of having stroke. An affirmative answer of “yes” was considered indicative of a stroke (Parikh et al. 2020; Cheng et al. 2023).

### 2.4 Covariates

To assess the influence of various factors on our results, we identified several potential confounding variables. These factors included age, sex (male or female), ethnicity such as Mexican American, white, black, or other, education level including less than high school, high school, or more than high school, marital status such as married or living with a partner, divorced, separated, widowed, or never married, income‐to‐poverty ratio, and vigorous recreational activity.

Smoking status is defined as never (smoked less than 100 cigarettes in a lifetime), former (used to smoke at least 100 cigarettes but has currently quit), or current (smoked more than 100 cigarettes in a lifetime and was still smoking at the time of the survey). Drinking status was classified as never (total alcohol consumption less than 12 drinks), former (total alcohol consumption more than 12 drinks, but no longer drinking), or current (more than 12 drinks per year). Body mass index (BMI) and waist circumference are derived from body measurements. BMI is calculated by dividing the measured weight by the square of the height. Data from laboratory blood tests consisted of WBC, lymphocyte count, monocyte count, neutrophil count, fasting glucose, glycated hemoglobin (HBA1c), and C‐reactive protein (CRP). In addition, we considered self‐reported chronic diseases such as hyperlipidemia, coronary heart disease (CHD), chronic kidney disease (CKD), diabetes, and hypertension, as well as the use of anti‐hypertensive, anti‐hyperlipidemic, and anti‐diabetic medications. By including these variables, we aimed to control for potential confounders, thereby enhancing the robustness and reliability of our findings.

### 2.5 Statistical Analysis

In this research, the study data were weighted in accordance with the NCHS guidelines. Missing data were imputed using predictive mean matching for numeric variables and logistic regression for binary variables (Liang et al. 2020). For baseline analysis, all participants were categorized into four groups according to their NPAR quartiles. Categorical variables were presented as percentages, while continuous variables were shown as means with standard errors. Statistical comparisons were conducted using weighted linear regression for continuous variables and weighted chi‐square tests for categorical variables. Since stroke was a dichotomous variable, logistic regression equations were used. This study developed three multivariate logistic regression models and restricted cubic splines to explore the association between NPAR and stroke. Model 1 made no adjustment for covariates, Model 2 adjusted only for age, gender, and race, and Model 3 adjusted for all covariates. Recursive algorithms and two‐stage linear regression models were used to determine the inflection point. Stratification and interaction analyses were performed to explore subgroup differences. Finally, we performed sensitivity analyses to confirm the stability of the findings. The study was statistically analyzed using R software (Version 4.0.2) and free statistics analysis platform, and a *p* value of less than 0.05 (two‐sided) was considered statistically significant.

## 3 Results

### 3.1 Baseline Characteristics of the Study Population

**FIGURE 2:**
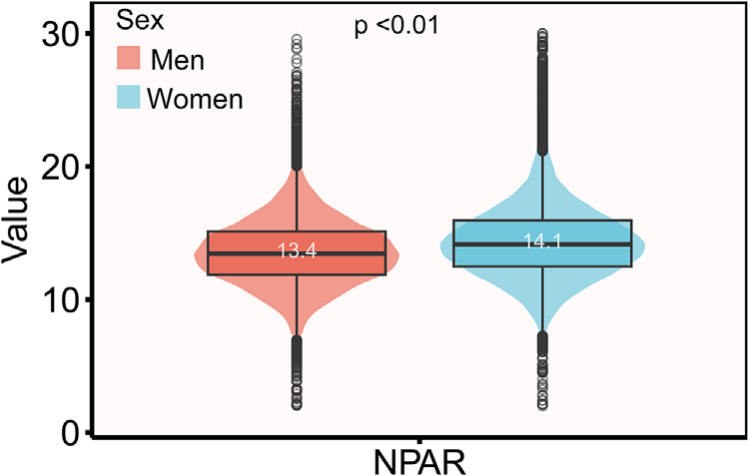
Sex differences in the distribution of neutrophil percentage‐to‐albumin ratio among men and women.

A total of 56,566 participants were selected for this study, and their baseline characteristics are shown in Table 1. Statistically, the mean age of all participants was 47.08 years old, of which 51.74% were female. Moreover, the NPAR values of males and females were compared and the mean NPAR for the females was 14.1, which was higher than that for males, and the difference between the two groups was statistically significant (*p* < 0.01) (**Figure** **2**). In addition, compared to quartile 1 of NPAR, the other quartile groups had more females, Whites, former smokers and drinkers, those who participated in vigorous recreational activities, subjects taking antihypertensive, antihyperlipidemic, and antidiabetic drugs, and had higher levels of BMI and waist. The prevalence of CHD, CKD, stroke, diabetes, and hypertension tended to increase with increasing NPAR. Furthermore, the baseline analysis of blood tests showed that the quartile 4 of NPAR had higher WBC, neutrophils, neutrophils percent, CRP, fasting glucose, and HBA1c than the other quartile groups. All the variables selected for this study were statistically significant (*p* < 0.01).

**TABLE 1:** Baseline characteristics of the study population from NHANES 1999–2020.

Variable	Total (*n* = 56,566)	Q1 (*n* = 14,157)	Q2 (*n* = 14,130)	Q3 (*n* = 14,137)	Q4 (*n* = 14,142)	*p*‐value
Age (years)	47.08 (0.17)	43.80 (0.24)	45.72 (0.21)	48.35 (0.23)	50.77 (0.23)	< 0.0001
Sex (%)						< 0.0001
Male	27,334 (48.26)	7972 (57.62)	7266 (51.96)	6632 (45.44)	5464 (36.98)	
Female	29,232 (51.74)	6185 (42.38)	6864 (48.04)	7505 (54.56)	8678 (63.02)	
Race/ethnicity (%)						< 0.0001
Mexican American	9611 (8.22)	2110 (7.64)	2623 (8.85)	2544 (8.44)	2334 (7.85)	
White	24,506 (68.08)	4850 (61.73)	6092 (68.66)	6616 (71.06)	6948 (70.95)	
Black	11,798 (10.66)	4356 (16.40)	2594 (8.87)	2341 (8.13)	2507 (9.35)	
Other	10,651 (13.04)	2841 (14.22)	2821 (13.62)	2636 (12.37)	2353 (11.85)	
Education level (%)						< 0.0001
Less than high school	6534 (5.75)	1551 (5.75)	1625 (5.59)	1733 (5.82)	1625 (5.86)	
High school	14,458 (22.24)	3553 (21.07)	3476 (21.02)	3564 (22.19)	3865 (24.96)	
More than high school	35,574 (72.01)	9053 (73.18)	9029 (73.39)	8840 (72.00)	8652 (69.18)	
Marital status (%)						< 0.0001
Married/Living with partner	34,098 (63.83)	8487 (63.72)	8793 (65.50)	8664 (64.83)	8154 (60.91)	
Divorced/Separated/Widowed	12,541 (18.39)	2666 (14.47)	2858 (16.87)	3221 (19.18)	3796 (23.49)	
Never married	9927 (17.78)	3004 (21.81)	2479 (17.63)	2252 (16.00)	2192 (15.60)	
Smoking status (%)						< 0.0001
Never	31,024 (54.12)	8076 (55.71)	7986 (55.41)	7646 (53.78)	7316 (51.28)	
Former	14,005 (24.90)	3226 (24.09)	3367 (24.31)	3597 (25.24)	3815 (26.10)	
Current	11,537 (20.98)	2855 (20.20)	2777 (20.28)	2894 (20.98)	3011 (22.62)	
Drinking status (%)						< 0.0001
Never	8313 (11.50)	2047 (11.38)	2005 (11.14)	2036 (11.33)	2225 (12.22)	
Former	8647 (12.93)	1890 (10.99)	2014 (11.82)	2135 (13.19)	2608 (16.02)	
Current	39,606 (75.58)	10,220 (77.63)	10,111 (77.05)	9966 (75.48)	9309 (71.76)	
Vigorous recreational activities (%)						< 0.0001
Yes	30,566 (47.23)	6832 (41.13)	7162 (43.78)	7711 (48.24)	8861 (56.72)	
No	26,000 (52.77)	7325 (58.87)	6968 (56.22)	6426 (51.76)	5281 (43.28)	
Hyperlipidemia (%)						< 0.0001
Yes	15,876 (29.11)	4424 (32.96)	3996 (29.45)	3803 (27.23)	3653 (26.65)	
No	40,690 (70.89)	9733 (67.04)	10,134 (70.55)	10,334 (72.77)	10,489 (73.35)	
CHD (%)						< 0.0001
Yes	2417 (3.58)	399 (2.49)	465 (2.72)	654 (3.41)	899 (5.94)	
No	54,149 (96.42)	13,758 (97.51)	13,665 (97.28)	13,483 (96.59)	13,243 (94.06)	
CKD (%)						< 0.0001
Yes	10,632 (14.48)	1804 (9.51)	2135 (11.52)	2706 (15.02)	3987 (22.67)	
No	45,934 (85.52)	12,353 (90.49)	11,995 (88.48)	11,431 (84.98)	10,155 (77.33)	
Stroke (%)						< 0.0001
Yes	2292 (2.93)	340 (1.63)	450 (2.23)	643 (3.19)	859 (4.83)	
No	54,274 (97.07)	13,817 (98.37)	13,680 (97.77)	13,494 (96.81)	13,283 (95.17)	
Diabetes (%)						< 0.0001
Yes	14,145 (20.09)	2865 (15.26)	3108 (16.69)	3603 (20.53)	4569 (28.77)	
No	42,421 (79.91)	11,292 (84.74)	11,022 (83.31)	10,534 (79.47)	9573 (71.23)	
Hypertension (%)						< 0.0001
Yes	23,376 (36.22)	5187 (31.10)	5423 (33.19)	6074 (37.90)	6692 (43.39)	
No	33,190 (63.78)	8970 (68.90)	8707 (66.81)	8063 (62.10)	7450 (56.61)	
Anti‐hypertensive drug (%)						< 0.0001
Yes	17,566 (26.11)	3592 (20.46)	3897 (22.76)	4571 (27.32)	5506 (34.76)	
No	39,000 (73.89)	10,565 (79.54)	10,233 (77.24)	9566 (72.68)	8636 (65.24)	
Anti‐hyperlipidemic drug (%)						< 0.0001
Yes	10,501 (16.41)	2153 (13.16)	2314 (14.07)	2877 (17.88)	3157 (21.02)	
No	46,065 (83.59)	12,004 (86.84)	11,816 (85.93)	11,260 (82.12)	10,985 (78.98)	
Anti‐diabetic drug (%)						< 0.0001
Yes	6351 (8.14)	1156 (5.19)	1315 (6.41)	1644 (8.25)	2236 (13.23)	
No	50,215 (91.86)	13,001 (94.81)	12,815 (93.59)	12,493 (91.75)	11,906 (86.77)	
BMI (kg/m^2^)	28.87 (0.06)	27.35 (0.07)	28.24 (0.08)	29.21 (0.10)	30.87 (0.10)	< 0.0001
Waist (cm)	98.74 (0.15)	94.81 (0.20)	97.26 (0.21)	99.79 (0.25)	103.54 (0.22)	< 0.0001
Income‐to‐poverty ratio	2.99 (0.02)	3.03 (0.03)	3.07 (0.03)	3.01 (0.03)	2.85 (0.03)	< 0.0001
WBC (1000 cells/uL)	7.29 (0.02)	6.44 (0.04)	6.94 (0.02)	7.46 (0.03)	8.42 (0.03)	< 0.0001
Lymphocyte (1000cells/uL)	2.14 (0.01)	2.55 (0.02)	2.19 (0.01)	2.01 (0.01)	1.79 (0.01)	< 0.0001
Monocyte (1000cells/uL)	0.57 (0.00)	0.56 (0.00)	0.56 (0.00)	0.57 (0.00)	0.58 (0.00)	< 0.0001
Neutrophils (1000 cells/uL)	4.33 (0.02)	3.06 (0.02)	3.94 (0.02)	4.64 (0.02)	5.82 (0.03)	< 0.0001
Fasting glucose (mg/dL)	104.74 (0.19)	101.45 (0.28)	103.01 (0.25)	105.36 (0.34)	109.61 (0.42)	< 0.0001
HBA1c (%)	5.58 (0.01)	5.49 (0.01)	5.52 (0.01)	5.61 (0.01)	5.73 (0.01)	< 0.0001
Neutrophils percent	58.53 (0.08)	47.72 (0.08)	56.57 (0.06)	62.03 (0.07)	68.58 (0.08)	< 0.0001
Serum albumin (g/dL)	4.26 (0.00)	4.44 (0.01)	4.35 (0.00)	4.24 (0.00)	3.97 (0.00)	< 0.0001
CRP (mg/dL)	1.66 (0.03)	0.89 (0.03)	1.16 (0.03)	1.56 (0.04)	3.20 (0.12)	< 0.0001

Abbreviations: BMI, body mass index; CHD, coronary heart disease; CKD, chronic kidney disease; CRP, C‐reactive protein; WBC, white blood cell.

### 3.2 Association Between NPAR and Stroke

In this study, three logistic regression models were constructed to analyze the association between NPAR and stroke, with the odds ratios (OR) and 95% confidence intervals (95% CI) presented in Table 2. In Model 1, without adjusting variables, the OR was 1.15 (95% CI: 1.13–1.17). Model 3, which adjusted for all confounding variables, showed an OR of 1.09 (95% CI: 1.05–1.12). This indicated that NPAR remained an independent risk factor for stroke even though the adjusted association was attenuated. In addition, through trend testing, we observed that compared to the first quartile of NPAR as a reference, the ORs and 95% CIs for the association between other quartiles of NPAR and stroke were: Q2 [1.34 (95% CI: 1.11–1.62)], Q3 [1.61 (95% CI: 1.31—1.97)], and Q4 [1.91 (95% CI: 1.50–2.43)].

**TABLE 2:** Association between neutrophil percentage‐to‐albumin ratio and stroke.

	Model 1 OR (95% CI)	Model 2 OR (95% CI)	Model 3 OR (95% CI)
NPAR	1.15 (1.13, 1.17)	1.09 (1.07, 1.12)	1.09 (1.05, 1.12)
NPAR category			
Q1	1.0	1.0	1.0
Q2	1.38 (1.14, 1.66)	1.27 (1.06, 1.54)	1.34 (1.11, 1.62)
Q3	1.99 (1.65, 2.39)	1.55 (1.29, 1.87)	1.61 (1.31, 1.97)
Q4	3.06 (2.55, 3.68)	1.98 (1.63, 2.41)	1.91 (1.50, 2.43)
Sex			
Men	1.24 (1.20, 1.27)	1.09 (1.06, 1.12)	1.08 (1.03, 1.12)
Women	1.09 (1.07, 1.12)	1.09 (1.06, 1.13)	1.08 (1.04, 1.13)

*Note*: Model 1: no covariates were adjusted.

Model 2: age, sex, and race were adjusted.

Model 3: age, sex, race, education level, income‐to‐poverty ratio, marital status, smoking status, drinking status, vigorous recreational activity, BMI, waist circumference, stroke, CKD, CHD, hyperlipidemia, diabetes, hypertension, anti‐hypertensive drug, anti‐hyperlipidemic drug, anti‐diabetic drug, WBC, lymphocyte, monocyte, neutrophils, HbA1c, fasting glucose, and CRP were adjusted.

**FIGURE 3:**
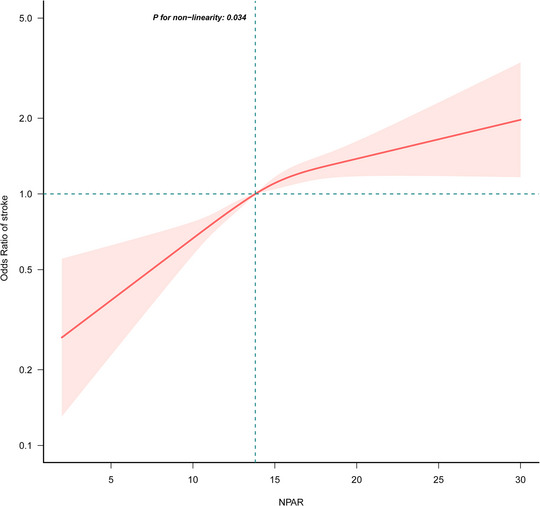
Association between neutrophil percentage‐to‐albumin ratio and stroke. Age, sex, race, education level, income‐to‐poverty ratio, marital status, smoking status, drinking status, vigorous recreational activity, BMI, waist circumference, stroke, CKD, CHD, hyperlipidemia, diabetes, hypertension, anti‐hypertensive drug, anti‐hyperlipidemic drug, anti‐diabetic drug, WBC, lymphocyte, monocyte, neutrophils, HbA1c, fasting glucose, and CRP were adjusted.

After adjusting for all confounding variables, restricted cubic splines were plotted to further clarify the association between NPAR and stroke (**Figure** **3**). The results revealed a nonlinear positive correlation between NPAR and stroke. It is worth noting that an inflection point (NPAR = 13.81) was observed, with ORs (95% CI) of 1.13 (1.08–1.17) before the inflection point and 1.04 (1.01–1.07) after the inflection point. This implies a consistently positive correlation between NPAR and stroke; however, beyond the inflection point, the increasing trend in stroke risk with higher NPAR levels begins to slow down (Table 3).

**TABLE 3:** Threshold effect analysis of neutrophil percentage‐to‐albumin ratio on stroke using a two‐piecewise linear regression model.

	Adjust OR (95% CI)	*p*‐value
Fitting by standard linear model	1.09 (1.05, 1.12)	< 0.0001
Fitting by two‐piecewise linear model		
Inflection point	13.81	
< 13.81	1.13 (1.08, 1.17)	< 0.0001
> 13.81	1.04 (1.01, 1.07)	0.0030
Log‐likelihood ratio	0.003	

*Note*: Age, sex, race, education level, income‐to‐poverty ratio, marital status, smoking status, drinking status, vigorous recreational activity, BMI, waist circumference, stroke, CKD, CHD, hyperlipidemia, diabetes, hypertension, anti‐hypertensive drug, anti‐hyperlipidemic drug, anti‐diabetic drug, WBC, lymphocyte, monocyte, neutrophils, HbA1c, fasting glucose, and CRP were adjusted.

### 3.3 Sex Differences in the Association Between NPAR and Stroke

**FIGURE 4:**
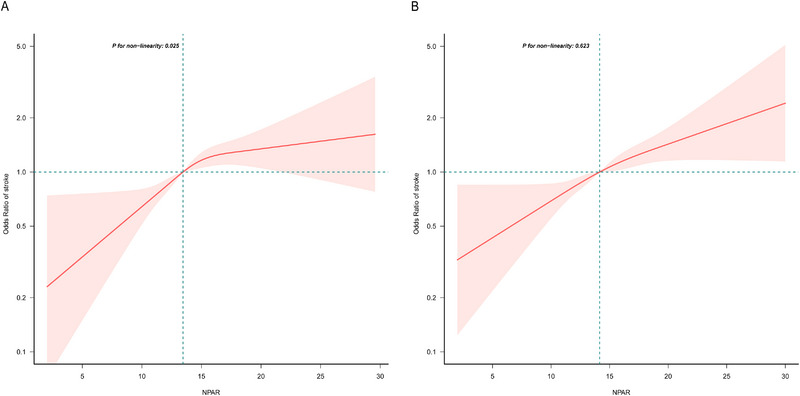
Association between neutrophil percentage‐to‐albumin ratio and stroke stratified by sex (A: men; B: women).

Stratified analyses by sex revealed that NPAR was positively correlated with stroke risk in both men and women (Table 2). Moreover, by plotting the restricted cubic splines and forest plots, the analysis indicated a nonlinear positive correlation between NPAR and stroke in men, whereas in women, the correlation was linear and positive (**Figures** **4** **and** **5**). In addition, to explore the potential association between NPAR and stroke in different populations, this study conducted subgroup analyses. No significant differences were found in the age, race, and BMI subgroups. The same results were observed in the lifestyle and chronic disease subgroups (**Figure** **6**).

**FIGURE 5:**
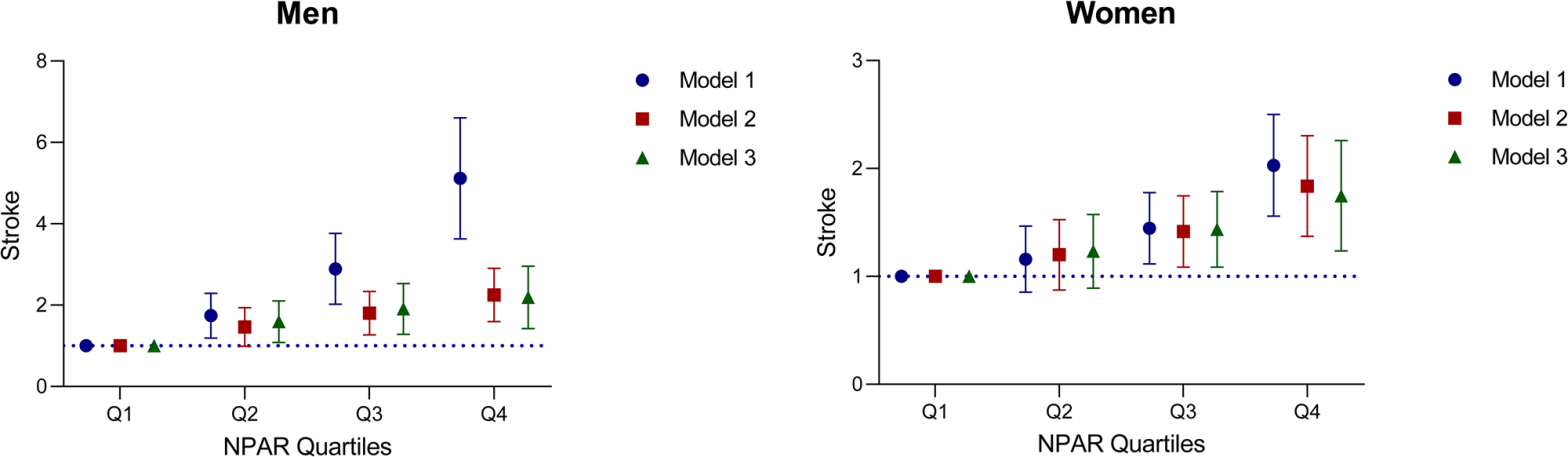
Association between quartiles of neutrophil percentage‐to‐albumin ratio and stroke.

**FIGURE 6:**
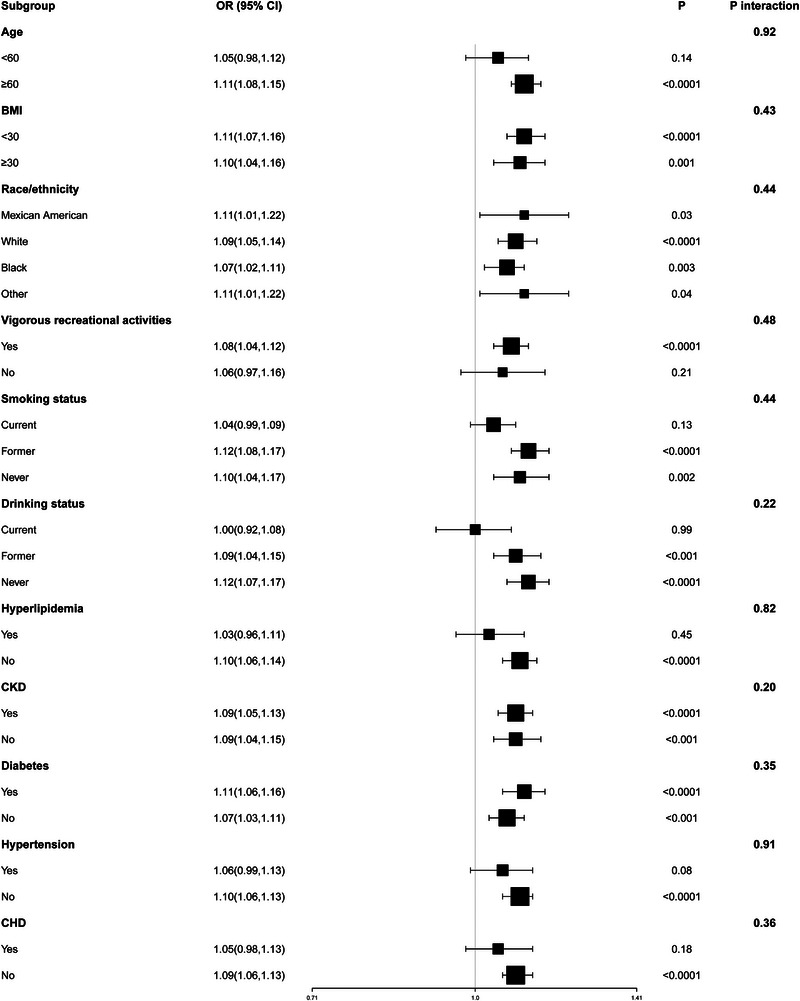
Subgroup analysis of risk factors for the relationship between neutrophil percentage‐to‐albumin ratio and stroke.

### 3.4 Sensitivity Analysis

To further explore the stability of the study results before and after interpolation, we conducted sensitivity analyses. No significant differences in the results before and after interpolation were observed in the study population characteristics (**Table** **S1**). Further multivariable logistic regression equations and restricted cubic sample plots also fully revealed the stability of the study results (Table **S2** and **S3** **;** **Figure** **S1**). Subgroup analyses likewise did not reveal the presence of significant interactions (**Figure** **S2**).

## 4 Discussion

To our knowledge, this is the first study exploring the association between NPAR and stroke. In this cross‐sectional study based on the American population, a nonlinear positive association between NPAR and stroke was found, characterized by a turning point and segmental effects. As NPAR increased, the probability of stroke occurrence gradually rose, but the rate of increase slowed down after the turning point. Furthermore, sex‐stratified analysis revealed a nonlinear positive association between NPAR and stroke in men and a linear positive association in women.

Inflammation is essential in the onset and progression of stroke, rendering inflammatory markers essential predictors of patient outcomes. Various lines of evidence indicate that inflammation causally contributes to the development of stroke (Kelly, Lemmens, and Tsivgoulis 2021; Candelario‐Jalil, Dijkhuizen, and Magnus 2022). This inflammatory response entails the activation and migration of immune cells, including neutrophils, macrophages, and lymphocytes, into the affected cerebral tissue (Kelly, Lemmens, and Tsivgoulis 2021). Although this response aims to clear debris and promote healing, it can also exacerbate cerebral injury through the release of cytokines, ROS, and proteases. The NPAR is a novel marker reflecting systemic inflammation and nutritional status, both critical in stroke pathology. An elevated NPAR, resulting from a higher neutrophil percentage and/or lower albumin levels, is associated with poorer outcomes in stroke patients (C. Xu et al. 2023). A significant amount of evidence suggests that neutrophils play a role in disrupting the blood–brain barrier (BBB), contributing to brain edema and cerebral injury (Jickling et al. 2015). This disruption exacerbates the imbalance between the immune and nervous systems, triggering both a localized inflammatory response and a systemic inflammatory reaction (Shi et al. 2018). Albumin plays several essential roles, such as functioning as a main buffer, detoxification agent, extracellular antioxidant, and immune regulator. Animal experiments have shown that albumin significantly enhances neurological function in models of middle cerebral artery occlusion (MCAO) and reduces infarction volume and brain swelling (Belayev et al. 2001; Dong et al. 2023). Research has shown that reduced serum albumin levels increase the risk of infectious complications (Thuemmler et al. 2024). Therefore, combining these two parameters into the NPAR could provide a more comprehensive indicator of the condition of stroke patients.

Research on the relationship between NPAR and stroke is relatively limited, previous studies have predominantly focused on either neutrophil count or serum albumin individually. A post hoc analysis of the CHANCE trial found that higher neutrophil counts and neutrophil ratios were significantly associated with an increased risk of new stroke, composite vascular events, and ischemic stroke (Zhu, Pan, et al. 2018). This research team also indicated that higher neutrophil counts, particularly in the presence of intracranial artery stenosis (ICAS), significantly elevated the risk of recurrent stroke and composite vascular events in patients with minor ischemic stroke (Zhu, Liu, et al. 2018). Moreover, a study conducted in the China Stroke Primary Prevention Trial (CSPPT) found that higher neutrophil counts were correlated with an increased risk of first stroke, especially in participants with high mean arterial pressure (MAP) or low total homocysteine (tHcy) levels (Zhang et al. 2021). In a retrospective cohort study of Chinese individuals, higher neutrophil counts and longitudinal increases exceeding 25% are tied to a significantly higher risk of fatal stroke (Z. Hu et al. 2021). In terms of the relationship between serum albumin and stroke, several studies have highlighted its significance. A study using data from the Korean National Health Insurance Service database showed that lower serum albumin levels are associated with an increased risk of stroke, including acute ischemic stroke and transient ischemic attack (Wang et al. 2023). Similarly, in a cohort study that included middle‐aged Japanese men and women, lower serum albumin levels are connected with an increased risk of total stroke (Li et al. 2021). A 12‐year prospective study involving 2,986 participants indicated that low serum albumin levels strongly predict the occurrence of a first stroke (W. Xu et al. 2014). In a recent prospective study that tracked approximately 100,000 individuals over 8.5 years, low serum albumin levels are independently associated with the occurrence of ischemic stroke. This association remained significant even after adjusting for BMI, inflammatory markers, liver and renal function, and other common risk factors (Ronit et al. 2020). Yang et al. 2024 reported that NPAR was independently associated with recurrence within 3 months in patients experiencing their first episode of acute ischemic stroke. Whereas, the further restricted cubic spline in our study, revealed that the positive correlation between NPAR and stroke was nonlinear.

Recent research has demonstrated that females have a greater lifetime risk of stroke, largely attributed to their longer life expectancy (Seshadri et al. 2006; Reeves et al. 2008). There are multiple reasons for sex differences in stroke incidence. Research indicates that women have an elevated risk of stroke due to factors such as pregnancy, the use of oral contraceptives, migraines, autoimmune conditions, and a higher risk of embolism in atrial fibrillation, with the protective effects of sex hormones waning after menopause (Poorthuis et al. 2017; Chang et al. 2018). In addition, variations in the inflammatory response to acute stroke between sexes, including BBB disruption and subsequent secondary ischemic, hemorrhagic, and edema‐related damage, contribute to these disparities (Tariq, Lee, and McCullough 2023). Estrogen is well‐known to exert a protective effect in females by modulating the immune response, specifically by reducing neutrophil infiltration at the site of stroke through the downregulation of chemokine activity. Research indicates that estrogen significantly inhibits neutrophil recruitment to stroke sites by suppressing the expression of chemoattractant‐2, a key chemokine involved in neutrophil chemotaxis (Miller et al. 2004). In aged mice, males exhibited a significantly higher number of brain neutrophils and elevated plasma concentrations of MCP‐1 and G‐CSF compared to females. This, combined with an increase in CD8+ T cells and Tregs, resulted in a 25% rise in mortality and a 55% higher risk of hemorrhagic transformation among male mice (Ahnstedt et al. 2018). Albumin also shows sex‐specific influences on stroke outcomes. Researchers have discovered that higher albumin levels are linked to a reduced incidence of cognitive impairment, especially in older men (Y. Hu et al. 2024). However, to date, no sex differences in this indicator have been observed in stroke.

This study leveraged a large‐scale sample of adults from the United States, ensuring both credibility and reproducibility of the data. To the best of our knowledge, this research is the first to investigate the link between NPAR and stroke, with the association carrying significant clinical and public health implications. Clinically, NPAR serves as a convenient and cost‐effective tool that can be integrated into routine blood tests to identify individuals at high risk for stroke, providing a basis for early intervention. In terms of public health, the application of NPAR can help optimize stroke prevention strategies, particularly in resource‐limited areas, by focusing on systemic inflammation and nutritional status to reduce stroke incidence. Moreover, this study identified sex differences in the relationship between NPAR and stroke, suggesting that future interventions may need to consider gender‐specific factors. Nevertheless, it is important to recognize several limitations. Firstly, the cross‐sectional design of the study prevents the establishment of causality, necessitating further prospective randomized controlled trials to validate these findings. Secondly, as participants were exclusively from the United States, the generalizability of the results to populations in other countries is uncertain. Thirdly, despite adjusting for potential confounders, residual confounding may still affect the observed association between NPAR and stroke. Lastly, the relevance of our findings to other populations, such as younger individuals, remains unclear and requires additional research.

## 5 Conclusion

In this cross‐sectional study of the American population, we discovered a nonlinear association between NPAR and stroke, and further studies identified sex differences in this association. The findings suggest the potential clinical significance of NPAR as a stroke risk marker, especially in the development of individualized stroke prevention and intervention measures. Future cohort studies are needed to validate this association and assess its value in clinical stroke prevention and prognosis.

## Author Contributions

**Shuying Xu**: writing–review and editing, writing–original draft. **Yiyan Sun**: data curation, writing–original draft. **Siyao Zhang**: investigation, writing–review and editing. **Yongjun Peng**: supervision, writing–review and editing, validation. **Dechun Dai**: writing–review and editing, funding acquisition, supervision. **Leiyong Zhao**: conceptualization, validation, writing–review and editing.

## Ethics Statement

The protocol was approved by the Institutional Review Board of National Center for Health Statistics (protocol number: Protocol#98‐12, Protocol#2005‐06, Continuation of Protocol #2005‐06, protocol#2011–17 and Continuation of Protocol #2011–17).

## Conflicts of Interest

The authors declare no conflicts of interest.

### Peer Review

The peer review history for this article is available at https://publons.com/publon/10.1002/brb3.70192.

## Supporting information



Supporting Information.

## Data Availability

The datasets used and/or analyzed during the current study are publicly available from the NHANES database, https://wwwn.cdc.gov/nchs/nhanes.
