# Supplementary material for: Relationship of Neutrophil Percentage‐to‐Albumin Ratio With Stroke: Evidence From NHANES 1999–2020
Source: Brain Behav. 2024 Dec 22;14(12):e70192. doi: 10.1002/brb3.70192 (PMC11664043; doi:10.1002/brb3.70192)

**Supplementary Table 1.** Baseline characteristics of the study

population before and after interpolation

| **Variable** | **Interpolation** | **Without**  **Interpolation** | **P-value** |
| --- | --- | --- | --- |
| Age (years) | 47.08 ± 16.95 | 47.08 ± 16.95 | 1.0000 |
| Sex (%) |  |  | 1.0000 |
| Male | 48.26 | 48.26 |  |
| Female | 51.74 | 51.74 |  |
| Race/ethnicity (%) |  |  | 1.0000 |
| Mexican American | 8.22 | 8.22 |  |
| White | 68.08 | 68.08 |  |
| Black | 10.66 | 10.66 |  |
| Other | 13.04 | 13.04 |  |
| Education level (%) |  |  | 0.9945 |
| Less than high school | 5.75 | 5.73 |  |
| High school | 22.24 | 22.24 |  |
| More than high school | 72.01 | 72.03 |  |
| Marital status (%) |  |  | 0.9994 |
| Married /Living with partner | 63.83 | 63.83 |  |
| Divorced/separated/widowed | 18.39 | 18.39 |  |
| Never married | 17.78 | 17.78 |  |
| Smoking status (%) |  |  | 0.9988 |
| Never | 54.12 | 54.13 |  |
| Former | 24.90 | 24.89 |  |
| Current | 20.98 | 20.98 |  |
| Drinking status (%) |  |  | 0.1023 |
| Never | 11.50 | 11.13 |  |
| Former | 12.93 | 13.19 |  |
| Current | 75.58 | 75.68 |  |
| Vigorous recreational activities (%) |  |  | 0.5349 |
| Yes | 47.23 | 47.14 |  |
| No | 52.77 | 52.86 |  |
| Hyperlipidemia (%) |  |  | 0.9886 |
| Yes | 29.11 | 29.11 |  |
| No | 70.89 | 70.89 |  |
| CHD (%) |  |  | 0.8689 |
| Yes | 3.58 | 3.55 |  |
| No | 96.42 | 96.45 |  |
| CKD (%) |  |  | 0.8367 |
| Yes | 14.48 | 14.40 |  |
| No | 85.52 | 85.60 |  |
| Diabetes (%) |  |  | 0.6895 |
| Yes | 20.09 | 20.27 |  |
| No | 79.91 | 79.73 |  |
| Hypertension (%) |  |  | 0.9947 |
| Yes | 36.22 | 36.22 |  |
| No | 63.78 | 63.78 |  |
| Anti-hypertensive drug (%) |  |  | 0.9947 |
| Yes | 36.22 | 36.22 |  |
| No | 63.78 | 63.78 |  |
| Anti-hyperlipidemic drug (%) |  |  | 0.9926 |
| Yes | 16.41 | 16.41 |  |
| No | 83.59 | 83.59 |  |
| Anti-diabetic drug (%) |  |  | 0.9769 |
| Yes | 8.14 | 8.14 |  |
| No | 91.86 | 91.86 |  |
| BMI (kg/m2) | 28.87 ± 6.78 | 28.87 ± 6.78 | 0.8488 |
| Waist (cm) | 98.74 ± 16.47 | 98.56 ± 16.35 | 0.0717 |
| Income-to-poverty ratio | 2.99 ± 1.64 | 3.00 ± 1.64 | 0.0678 |
| WBC (1000cells/ul) | 7.29 ± 2.66 | 7.29 ± 2.66 | 1.0000 |
| Lymphocyte (1000cells/ul) | 2.14 ± 1.63 | 2.14 ± 1.63 | 1.0000 |
| Monocyte (1000cells/ul) | 0.57 ± 0.19 | 0.57 ± 0.19 | 1.0000 |
| Neutrophils (1000cells/ul) | 4.33 ± 1.70 | 4.33 ± 1.70 | 1.0000 |
| Fasting glucose(mg/dl) | 104.74 ± 30.29 | 104.69 ± 31.09 | 0.6896 |
| HBA1c(%) | 5.58 ± 0.92 | 5.58 ± 0.92 | 0.9943 |
| CRP (mg/dl) | 1.66 ± 4.70 | 1.67 ± 4.79 | 0.7079 |

**Supplementary** **Table 2.** Association between neutrophil percentage-to-albumin ratio and stroke (Without interpolations for covariates)

|  | **Model 1 OR (95% CI)** | **Model 2 OR (95% CI)** | **Model 3 OR (95% CI)** |
| --- | --- | --- | --- |
| NPAR | 1.15(1.13,1.17) | 1.09(1.07,1.12) | 1.07 (1.05, 1.10) |
| NPAR category |  |  |  |
| Q1 | 1.0 | 1.0 | 1.0 |
| Q2 | 1.33 (1.16, 1.54) | 1.32 (1.14, 1.53) | 1.29 (1.11, 1.51) |
| Q3 | 1.93 (1.69, 2.21) | 1.67 (1.46, 1.92) | 1.57 (1.35, 1.83) |
| Q4 | 2.62 (2.31, 2.98) | 1.97 (1.73, 2.25) | 1.67 (1.41, 1.98) |
| Sex |  |  |  |
| Men | 1.20 (1.17, 1.22) | 1.09 (1.07, 1.12) | 1.07 (1.03, 1.10) |
| Women | 1.07 (1.05, 1.09) | 1.09 (1.07, 1.11) | 1.08 (1.04, 1.11) |

Model 1: no covariates were adjusted..

Model 2:.age, sex, race were adjusted..

Model 3:age, sex, race, education level, income-to-poverty ratio, marital status, smoking status, drinking status, vigorous recreational activity, BMI, waist circumference, stroke, CKD, CHD, hyperlipidemia, diabetes, hypertension, anti-hypertensive drug, anti-hyperlipidemic drug, anti-diabetic drug, WBC, lymphocyte, monocyte, neutrophils, HbA1c, fasting glucose, and CRP were adjusted.

**Supplementary Table 3.** Threshold effect analysis of neutrophil percentage-to-albumin ratio on stroke using a two-piecewise linear regression model (Without interpolations for covariates)

|  | **Adjust OR (95% CI)** | **P-value** |
| --- | --- | --- |
| Fitting by standard linear model | 1.07 (1.05, 1.10) | <0.0001 |
| Fitting by two-piecewise linear model |  |  |
| Inflection point | 13.81 |  |
| <13.81 | 1.13 (1.08, 1.18) | <0.0001 |
| >13.81 | 1.05 (1.02, 1.08) | 0.0021 |
| Log-likelihood ratio | 0.003 |  |

Age, sex, race, education level, income-to-poverty ratio, marital status, smoking status, drinking status, vigorous recreational activity, BMI, waist circumference, stroke, CKD, CHD, hyperlipidemia, diabetes, hypertension, anti-hypertensive drug, anti-hyperlipidemic drug, anti-diabetic drug, WBC, lymphocyte, monocyte, neutrophils, HbA1c, fasting glucose, and CRP were adjusted.

**Supplementary Fig. 1** Association between neutrophil percentage-to-albumin ratio and stroke(Without interpolations for covariates; A: All; B: men ; C: women).


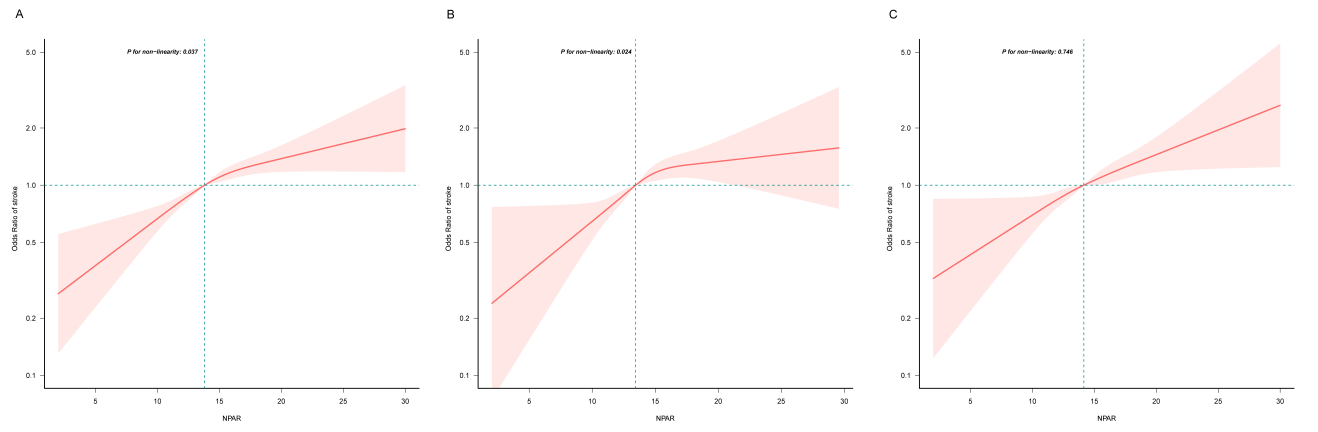


**Supplementary Fig. 2** Subgroup analysis of risk factors for the relationship between neutrophil percentage-to-albumin ratio and stroke (Without interpolations for covariates).


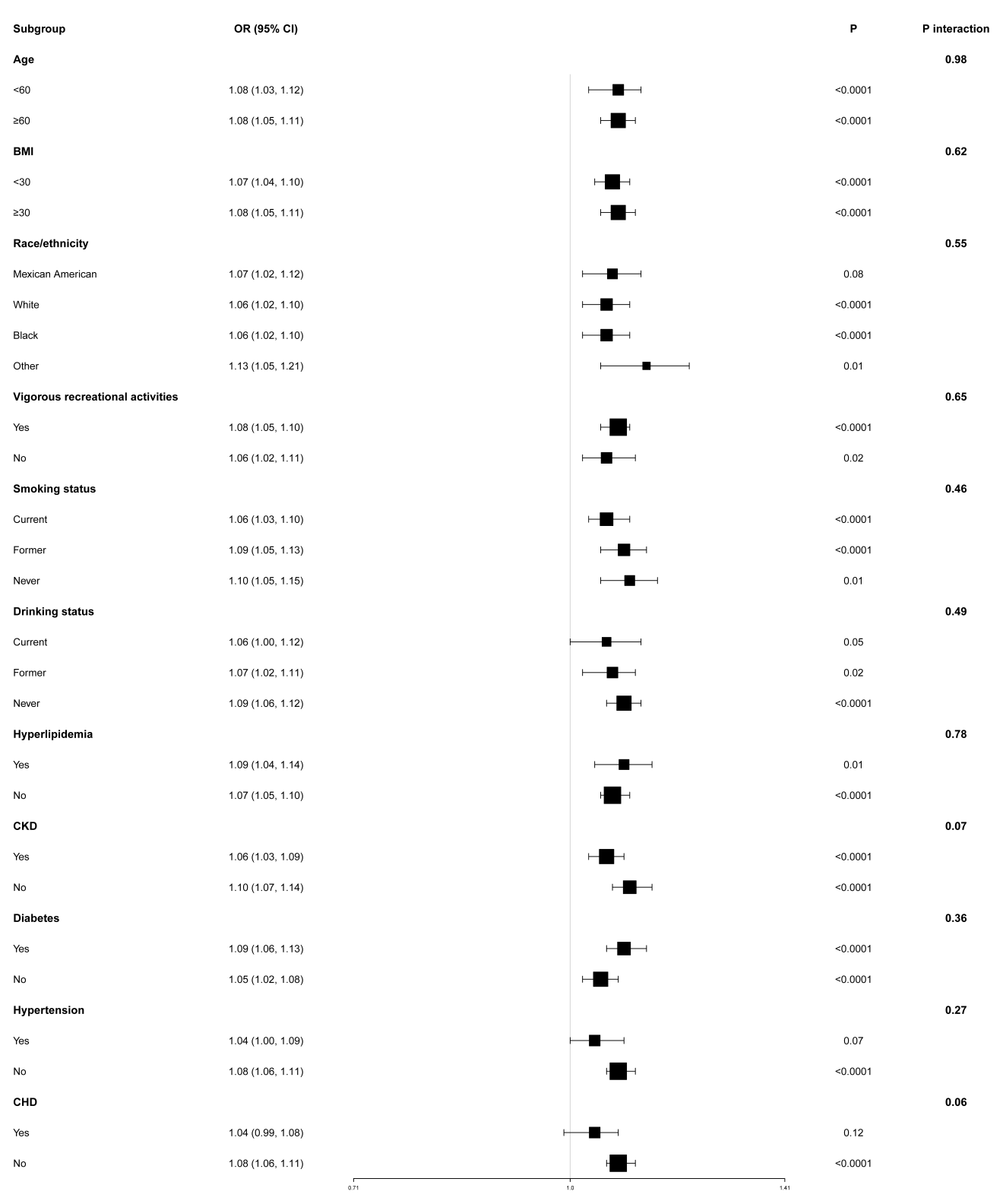

Supplement: Supplementary file 1 — Supporting Information. [file BRB3-14-e70192-s001.docx]
